# Pharmacokinetic, pharmacodynamic and biomarker evaluation of transforming growth factor-β receptor I kinase inhibitor, galunisertib, in phase 1 study in patients with advanced cancer

**DOI:** 10.1007/s10637-014-0192-4

**Published:** 2014-12-23

**Authors:** Jordi Rodón, Michael Carducci, Juan M. Sepulveda-Sánchez, Analía Azaro, Emiliano Calvo, Joan Seoane, Irene Braña, Elisabet Sicart, Ivelina Gueorguieva, Ann Cleverly, N. Sokalingum Pillay, Durisala Desaiah, Shawn T. Estrem, Luis Paz-Ares, Matthias Holdhoff, Jaishri Blakeley, Michael M. Lahn, Jose Baselga

**Affiliations:** 1 Medical Oncology, Vall d’Hebron University Hospital and Universitat Autonoma de Barcelona, Barcelona, Spain; 2 Johns Hopkins Kimmel Cancer Center, Baltimore, MD USA; 3 Hospital Universitario 12 de Octubre, Madrid, Spain; 4 Eli Lilly and Company, Erl Wood, UK; 5 Eli Lilly and Company, Indianapolis, IN USA; 6 Hospital Virgen del Rocío, Sevilla, Spain; 7 Neurology/Neurosurgery/and Oncology, Johns Hopkins University, Baltimore, MD USA; 8 Servei d’Oncologia Medica, Hospital Universitari Vall d’Hebron, Vall d’Hebron Institute of Oncology (V.H.I.O.), Passatge Vall d’Hebron 119, 08035 Barcelona, Spain; 9 Present Address: START Madrid, Centro Integral Oncológico Clara Campal, Madrid, Spain

**Keywords:** TGF-β inhibitor, Galunisertib, First-in-Human Dose, Glioma, Pharmacokinetics, Pharmacodynamics

## Abstract

*Purpose* Transforming growth factor-beta (TGF-β) signaling plays a key role in epithelial-mesenchymal transition (EMT) of tumors, including malignant glioma. Small molecule inhibitors (SMI) blocking TGF-β signaling reverse EMT and arrest tumor progression. Several SMIs were developed, but currently only LY2157299 monohydrate (galunisertib) was advanced to clinical investigation. *Design* The first-in-human dose study had three parts (Part A, dose escalation, *n* = 39; Part B, safety combination with lomustine, *n* = 26; Part C, relative bioavailability study, *n* = 14). *Results* A preclinical pharmacokinetic/pharmacodynamic (PK/PD) model predicted a therapeutic window up to 300 mg/day and was confirmed in Part A after continuous PK/PD. PK was not affected by co-medications such as enzyme-inducing anti-epileptic drugs or proton pump inhibitors. Changes in pSMAD2 levels in peripheral blood mononuclear cells were associated with exposure indicating target-related pharmacological activity of galunisertib. Twelve (12/79; 15 %) patients with refractory/relapsed malignant glioma had durable stable disease (SD) for 6 or more cycles, partial responses (PR), or complete responses (CR). These patients with clinical benefit had high plasma baseline levels of MDC/CCL22 and low protein expression of pSMAD2 in their tumors. Of the 5 patients with IDH1/2 mutation, 4 patients had a clinical benefit as defined by CR/PR and SD ≥6 cycles. Galunisertib had a favorable toxicity profile and no cardiac adverse events. *Conclusion* Based on the PK, PD, and biomarker evaluations, the intermittent administration of galunisertib at 300 mg/day is safe for future clinical investigation.

## Introduction

Transforming growth factor-beta (TGF-β) ligands (TGF-β1, TGF-β2, TGF-β3) regulate diverse biological functions [1, 2]. Any of these three ligands can engage the specific receptor TGF-βRI, which then heterodimerizes with TGF-βRII. This heterodimer complex phosphorylates the intracellular proteins SMAD2 and SMAD3 that activates a signaling cascade to induce several nuclear transduction proteins. With the induction of such proteins, the TGF-β signaling pathway influences cellular proliferation, differentiation, motility, survival and apoptosis in tumor cells. This can promote epithelial-mesenchymal transition (EMT) of a tumor, including malignant glioma, making TGF-β signaling a key driver of tumor progression [3, 4]. Patients with glioma who receive trabedersen, an antisense oligonucleotide designed to block TGF-β2 [5], show clinical benefit suggesting that blocking this pathway may result in anti-tumor activity.

Like trabedersen, the TGF-βRI kinase small molecule inhibitor (SMI) LY2157299 monohydrate (galunisertib) was developed to block the TGF-β signaling in cancer [6]. Because galunisertib is the first SMI to be clinically investigated in patients, a predictive pharmacokinetic/pharmacodynamic (PK/PD) model was developed to identify a safe therapeutic window for the first-in-human dose (FHD) study [7]. In the FHD study, the predictions from the PK/PD model were confirmed after PK profiles and PD assessments were obtained from patients in each cohort [8]. The PD assessments were based on a previously developed enzyme-linked immunosorbent assay (ELISA) that measured changes of the phosphorylated SMAD2 (pSMAD2) [9]. In addition, we evaluated other baseline biomarkers in plasma and in tumor tissue as future candidates for prognostic or predictive markers of TGF-β inhibitors [10]. Among these baseline markers, we determined whether the patients with IDH1/2 mutations had either radiographic responses or stable disease (SD). In addition, we investigated whether baseline high plasma MDC/CCL22 levels were associated with clinical benefit.

## Methods

### Patients

Patients were eligible to participate in the study if they had a histologic or cytologic diagnosis of cancer, progressed on previous therapies and had measurable tumors. Starting with Cohort 3, only patients with relapsed and progressive glioma were eligible for this study. All patients were assessed for radiographic responses using Response Evaluation Criteria in Solid Tumors (RECIST) and, for glioma patients, using Macdonald criteria [11]. All patients had to have an Eastern Cooperative Oncology Group performance status of ≤2. Patients were required to have adequate hematologic, hepatic, and renal function and discontinue all previous therapies for cancer at least 4 weeks prior to study enrollment. Exclusion criteria included medically uncontrolled cardiovascular illness, electrocardiogram anomalies, serious pre-existing medical conditions, and any unapproved therapy.

The study was conducted according to the principles of good clinical practice, applicable laws and regulations, and the Declaration of Helsinki. Each institution’s review board approved the study and all patients signed an informed consent document before study participation.

### Study design

Galunisertib was evaluated in a 3-part, multicenter, open-label, nonrandomized, dose-escalation first-in-human Phase I study.

- **Part A** was a dose escalation study using galunisertib monotherapy administered initially to patients with advanced or metastatic cancer as a daily continuous dosing. Starting with Cohort 3 and for the remainder of the study, patients received galunisertib on an intermittent dose regimen of 14 days on/14 days off for a 28-day cycle.
- **Part B** was a safety study using galunisertib on an intermittent dose regimen at 160 mg/day (80 mg twice daily [BID]) and 300 mg/day (150 mg BID) in combination with lomustine given once every 6 weeks in patients with recurrent malignant glioma who had previously failed approved treatments.
- **Part C** was a relative bioavailability (RBA) crossover study conducted at only one center to assess two new formulations (publication in preparation). All patients were then eligible to continue on 300 mg/day dosing. In this part of the study, patients with different tumor histologies were eligible to participate; the majority of patients had glioma. The only results from Part C presented in this publication are the results of the T cell subset examination. Other results will be published elsewhere.

### Study objectives

The objectives of this study were to characterize the PK profile of galunisertib monotherapy (Part A) and galunisertib in combination with lomustine (Part B). In addition, the relationship of pSMAD2 to galunisertib exposure was assessed (Part A). Biomarkers in plasma and tissue were sampled at baseline and during treatment to identify possible prognostic or predictive biomarkers.

### Treatment

In Part A, galunisertib was given orally BID at doses of 20 mg (40 mg/day), 40 mg (80 mg/day), 80 mg (160 mg/day), 120 (240 mg/day) and 150 mg (300 mg/day). In Part B, patients in Cohort 1 received galunisertib at 160 mg/day on intermittent dosing as defined in Part A. Lomustine 100 to 130 mg/m^2^ was given orally once every 6 weeks beginning 1 week after initial galunisertib dosing. In Cohort 2, 300 mg/day of galunisertib was given on an intermittent dosing schedule and lomustine as in Cohort 1. In Part C, all patients received 300 mg/day monotherapy of galunisertib.

### Bioanalytical methods

Human plasma samples obtained during this study were analyzed at PharmaNet USA, Inc. Princeton, New Jersey, USA. The samples were analyzed for galunisertib levels using 2 validated liquid chromatography with tandem mass spectrometry methods. For the high-range method (PharmaNet USA, Inc. SOP TM.589), the lower limit of quantification was 5.000 ng/mL and the upper limit of quantification was 1000.000 ng/mL. The intra-assay accuracy (% relative error) during validation ranged from 1.800 to 10.222 %. The intra-assay precision (% relative standard deviation) during validation ranged from 3.119 to 18.389 %. Samples above the limit of quantification were diluted to yield results within the calibrated range. Samples below the lower limit of quantification using the high-range method were reanalyzed using the low-range method. For the low-range method (PharmaNet USA, Inc. SOP TM.563), the lower limit of quantification was 0.050 ng/mL and the upper limit of quantification was 10.000 ng/mL. The intra-assay accuracy during validation ranged from −5.080 to −2.000 %. The intra-assay precision during validation ranged from 4.404 to 12.245 %.

### Pharmacokinetic methods

All patients who received at least 1 dose of galunisertib and had samples collected were subjected to PK analyses. The PK parameters for galunisertib were computed by standard non-compartmental methods of analysis using Win Nonlin Professional Edition (version 5.3) on a computer that met or exceeded the minimum system requirements for this program with appropriate and validated software. The parameters reported from the non-compartmental analyses included the maximum plasma concentration (C_max_), area under the curve from zero to 24 h (AUC_(0–24)_), AUC from zero time to infinity (AUC_(0-∞)_)_,_ half-life volume of distribution (V_d_) and clearance (CL).

### Pharmacodynamics pSMAD2 ELISA

pSMAD2 in peripheral blood mononuclear cells (PBMCs) were assessed by a specific ELISA as previously described [9], in which a rabbit polyclonal antibody recognizing pSMAD2 was used. Total SMAD2 was also assessed and used to normalize the expression of pSMAD2.

### Flow cytometry for T cell subsets

Blood samples were obtained and prepared for flow cytometry assessment following the instructions of Quintiles Laboratories (Durham, NC). After red blood cell lysis, cells were stained for CD3^+^, CD8^+^, CD4^+^ and CD4^+^CD25^+^ CD127^−^/LOFoxp3^+^ and events were collected by flow cytometer per standard Quintiles procedures. All results were reported as absolute cell counts and as percentage of lymphocytes.

### Baseline biomarker assessments

Plasma samples were analyzed for PD assessments by using a multi-analyte immunoassay panel (MAIP) (Myriad RBM, Austin, TX). The MAIP measurements included 89 plasma proteins [10]. These proteins were evaluated at baseline and during the first cycle of treatment.

Tumor tissue from the initial pathological diagnosis was obtained and slides for immunohistochemistry (IHC) staining were sent to Ventana (Tucson, AZ). All slides were stained for pSMAD2 (Ser465/467, 138D4, Rabbit mAb) following recommendation by the manufacturer (Cell Signaling, MA).

Genetic mutation assessments (including IDH1/2) were conducted at Foundation Medicine (New York, NY) following previously described sequencing and analysis approach [11]. DNA was extracted, sequenced, and 287 genes assessed for point mutations, short insertions/deletions, copy number alterations, and re-arrangements.

### Statistical analyses

A power model was fitted to PK parameter estimates C_max_ and AUC_(0–24)_ to assess the extent of dose proportionality. Clinical benefit in this study has been defined as a patient with either a complete response (CR), partial response (PR), and stable disease (SD) ≥6 cycles [8]. MAIP results from plasma samples taken at baseline were compared between patients completing >6 cycles and ≤6 cycles using ANOVA. The analysis included controlling the false discovery rate to 30 % using the Benjamini-Hochberg method. Boxplots showing the distributions at baseline of the potentially prognostic markers are provided. Observed TGF-β-stimulated pSMAD2 (pSMAD2^+)^ in PBMCs was normalized by TGF-β-stimulated tSMAD2 [(tSMAD2^+^) ^0.6^[9,10]] prior to determining the minimum normalized pSMAD2^+^ and calculating percentage inhibition post-baseline for each patient. Baseline and minimum post-baseline values for each patient together with maximum percentage inhibition are presented on a line and scatter plot for each patient in Cohort 3 and summary statistics by time point provided from a standard mixed effects regression model, fitting log normalized pSMAD2^+^ to the fixed effects of log baseline normalized pSMAD2^+^, nominal time point, dose and their interaction and patient as a random effect.

No statistical analyses were performed on IHC scores of pSMAD2 in tissue, T cells and lymphocytes. The former are summarized using box whisker plots and the latter are displayed in line and scatter plots.

## Results

### FHD study design and patient characteristics

In this three-part FHD study, 79 patients were enrolled. The details of the study design and patient demographics have been reported elsewhere [8], but a brief description is provided here. The study started in 2006 and was closed in 2012. In Part A (galunisertib monotherapy, dose-escalation), 39 patients were enrolled: in Cohort 1 (40 mg/day) and Cohort 2 (80 mg/day), 7 patients with advanced or metastatic cancer, and 32 patients with glioma in Cohorts 3, 4, and 5. In Part B (galunisertib combined with lomustine), 26 patients received galunisertib in combination with lomustine. In Part C (RBA followed by monotherapy), 14 patients (9 patients with glioma) completed the RBA study and elected to continue galunisertib treatment. Most patients with glioma had primary Grade IV glioblastoma (50 % in Part A, 76.9 % in Part B, not collected in Part C). However, in Part A there were more patients with secondary glioblastoma or low-grade glioma compared to Part B (Table 1). As of June 2014, 3 patients were still receiving galunisertib: 2 in Part A (treated for 73 and 55 cycles respectively, or 5 and 4.2 years, respectively) and 1 in Part B (treated for 48 cycles or 3.7 years).

**Table 1:** Patient characteristics

Characteristics	Part A *N* = 39	Part B *N* = 26	Part C *N* = 14
Age, years			
Mean (SD)	51.8 (14.88)	44.5 (10.35)	59.8
Median (Range)	54 (22–77)	43.5 (25–61)	56.5 (34–76)
Sex, n (%)			
Male	30 (76.9)	19 (73.1)	5 (35.7)
Female	9 (23.1)	7 (26.9)	9 (64.3)
ECOG, n (%)			
0	15 (38.5)	3 (11.5)	4 (28.6)
1	19 (48.7)	17 (65.4)	8 (57.1)
2	5 (12.8)	6 (23.1)	2 (14.3)
Number of prior regimens, n (%)			
1	17 (43.6)	7 (26.9)	2 (14.3)^a^
2	13 (33.3)	11 (42.3)	4 (28.6)^a^
3	4 (10.3)	7 (26.9)	2 (14.3)^a^
> 3	5 (12.8)	1 (3.8)	1 (7.1)^a^
Patients with glioma only	*N* = 32	*N* = 26	*N* = 9
Time from initial diagnosis to before first dose, median (range: earliest to most recent) in months	22.1 (172.4−2.8)	18 (154.6−7.0)	Not collected
Glioma WHO, at study entry, n (%)	*n* = 30	*n* = 26	Not collected
Grade I	1 (3.3)	–	
Grade II	2 (6.7)	–	
Grade III	6 (20)	4 (15.4)	
Grade IV	21 (70)	22 (84.6)	
Secondary grade IV	6 (20)	2 (7.7)	
Primary grade IV	15 (50)	20 (76.9)	
Tissue samples for deep sequencing	*n* = 11	*n* = 10	Not collected
IDH1/2 mutation, n (%)	3 (27.3)	2 (20)	

^a^Glioma patients only (Part C)

Abbreviations: *ECOG* eastern cooperative oncology group, *WHO* world health organization

### Pharmacokinetic measures

**Fig. 1:**
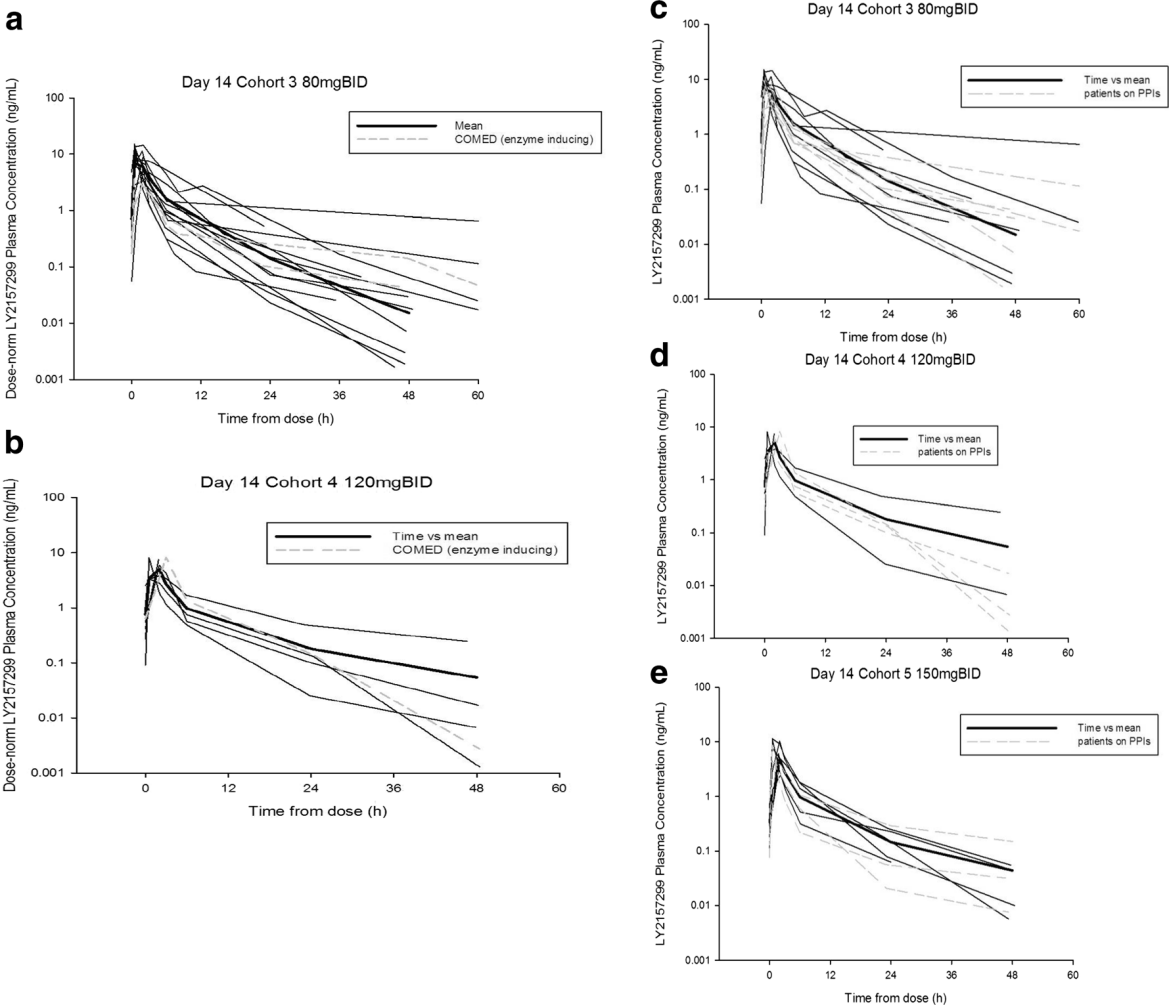
Pharmacokinetic profile of galunisertib at Day 14 (Part A) across doses of 80 mg BID, 120 mg BID and 150 mg BID. Co-medication of EIAE drugs (**panels a and b**): In Part A, 3 patients (2 in Cohort 3 and 1 in Cohort 4) received galunisertib while being on an EIAE medication. The PK profile of these patients (shown by broken grey lines) does not appear to differ from the other patients. Co-medication with PPIs (**panels c-e**): Plasma galunisertib individual concentration time profiles for all patients and patients on PPIs plotted on Day 14 following oral doses of 80 mg (160 mg/day), 120 mg (240 mg/day), and 150 mg (300 mg/day) BID. Fourteen patients (1 in Part A Cohort 2 [40 mg BID]; 6 in Part A Cohort 3 [80 mg BID], **panel c**; 4 in Part A Cohort 4 [120 mg BID], **panel d;** and 3 in Part A Cohort 5 [150 mg BID], **panel e**) received galunisertib while on a PPI medication. The PK profile of these patients (shown by broken grey lines) does not appear to differ from the other patients. Abbreviation: *BID* twice daily, *EIAE* enzyme-inducing anti-epileptic, *PK* pharmacokinetic, *PPI* proton pump inhibitors

- **Part A** (Fig. 1 and Table 2): Non-compartmental PK analysis was performed on 37 of the 39 patients treated in Part A: *n* = 3 in Cohort 1 (40 mg/day); *n* = 4 in Cohort 2 (80 mg/day); *n* = 15 in Cohort 3 (160 mg/day); *n* = 6 in Cohort 4 (240 mg/day); *n* = 9 in Cohort 5 (300 mg/day). Results showed rapid absorption of galunisertib, as demonstrated by measurable plasma concentrations for at least 48 h. The terminal half-life was approximately 8 h. At steady state, on Day 14, the median time to maximum concentration (t_max,ss_) ranged from 0.5 to 2 h post dose, independent of dose. Formal assessment of time-linear kinetics was not possible, regardless of whether or not the observed exposures of AUC_(0-∞)_ Day 1 and AUC_(0-∞)_ Day 14 were similar. However, no accumulation of galunisertib in the 5 cohorts was observed over the 14-day BID dosing regimen. Both the maximum observed plasma concentration at steady state (C_max,ss_) and exposure increased with dose as indicated from the statistical analysis of the PK parameters. The estimated ratios of geometric means for the AUC_(0-∞)_ and C_max,ss_ between 40 and 300 mg daily (7.5-fold) were 5.61 (90 % confidence intervals [CI]: 3.80, 8.30) and 3.99 (90 % CI: 2.43, 6.54), respectively. For a doubling of dose, the fold increases for AUC_(0-∞)_ and C_max,ss_ were 1.81-fold with corresponding 90 % CI (1.58, 2.07) and 1.61-fold with 90 % CI (1.36, 1.91), respectively. This suggests dose-proportional PK for any doubling of dose within the studied dose range of 40–300 mg, particularly for AUC_(0-∞)_. Within- and between-patient coefficients of variation were estimated as 29 and 42 %, respectively, for AUC_(0-∞)_ at steady state, and 31 and 55 %, respectively, for C_max,ss_, all pooled across the 5 cohorts from the dose-proportionality analysis. A population PK model was developed based on data in Cohorts 1 through 5 [7]. The mean population CL of galunisertib was 38 L/h, and the steady state volume of distribution (V_ss_) was 210 L.

**Table 2:** Galunisertib plasma pharmacokinetic data and pharmacodynamic changes in patients from Part A

Total dose (mg/day)	Number of patients cycle 1–2	Observations mean (% CV), day 14, steady state			
		Cycle 1		Cycle 2	
		C_max, ss_ (ng/mL)	AUC_0,∞_ (ng*h/mL)	C_max, ss_ (ng/mL)	AUC_0,∞_ (ng*h/mL)
40	3 - NA	220 (92)	518* (60)	NA	NA
80	4 - NA	350 (44)	1310* (41)	NA	NA
160	13−9	630 (58)	2140 (52)	790 (51)	2430 (33)
240	5−2	660 (44)	3060 (49)	520-^a^ 610-^a^	2900-^a^ 2500-^a^
300	9−7	990 (57)	3730 (46)	800 (58)	2930 (63)
Percent Inhibition of pSMAD2 (normalized^b^) at the 160 mg/day cohort (Part A): Observed, fitted Percentage Inhibition of Normalized^b^ pSMAD2 – (N), (95 % CI)					
Day 1				Day 12 or 15	
0.5	4	6		Post Dose	
26 %, 27 % (13), (−43, 62)	5 %, 5 % (14), (−82, 51)	−10 %, −10 % (14), (−112, 43)		39 %, 34 % (15), (−26, 65)	

Abbreviations: *CI* confidence interval, *h* hour, *pSMAD* phosphorylated SMAD2, *tSMAD* total SMAD

^a^Individual parameters (n <3), ^b^pSMAD2 is normalized by dividing pSMAD2^+^ by tSMAD2^+^ to the power of 0.6. Note: The time point “Post dose, Day 12/15” includes 2, 3, 4, 6 h Day 12 for 160 mg. The fitted results are derived from the mixed-effects model
- **Part B**: Patients were administered lomustine in combination with galunisertib. PK profiles of galunisertib following administration of 160 mg/day (Cohort 6) and 300 mg/day (Cohort 7) on Days 6 and 7 were similar. Hence, co-administration of lomustine did not appear to alter the galunisertib PK profile.

### Pharmacokinetics in patients receiving enzyme-inducing and nonenzyme-inducing anti-epileptic drugs and proton pump inhibitors

Patients with glioblastoma received several drugs that help control epileptic events, specifically carbamazepine, felbamate, oxcarbazepine, phenobarbital, phenytoin, and topiramate. These drugs are known to alter PK profile of therapeutic agents, especially if such agents are metabolized via the liver. One such example has been reported on imatinib in the treatment of glioblastoma [12]. In this study, 3 patients (2 in Cohort 3 and 1 in Cohort 4) received galunisertib while on an enzyme-inducing medication. The PK profiles of these patients (shown by broken grey lines in Fig. 1a and b) do not appear to differ from the other patients. Additionally, PK profiles of patients who were on proton pump inhibitors (PPIs) were plotted together with remaining patients to investigate any influence on galunisertib exposure. The most common PPI prescribed to patients was omeprazole. Fourteen patients (1 in Cohort 2, 6 in Cohort 3, 4 in Cohort 4, and 3 in Cohort 5) received galunisertib while on a PPI medication. The PK profiles of these patients (shown by broken grey lines in Fig. 1c, d, and e) do not appear to be altered by co-administration with PPIs.

### Pharmacodynamic evaluation

**Fig. 2:**
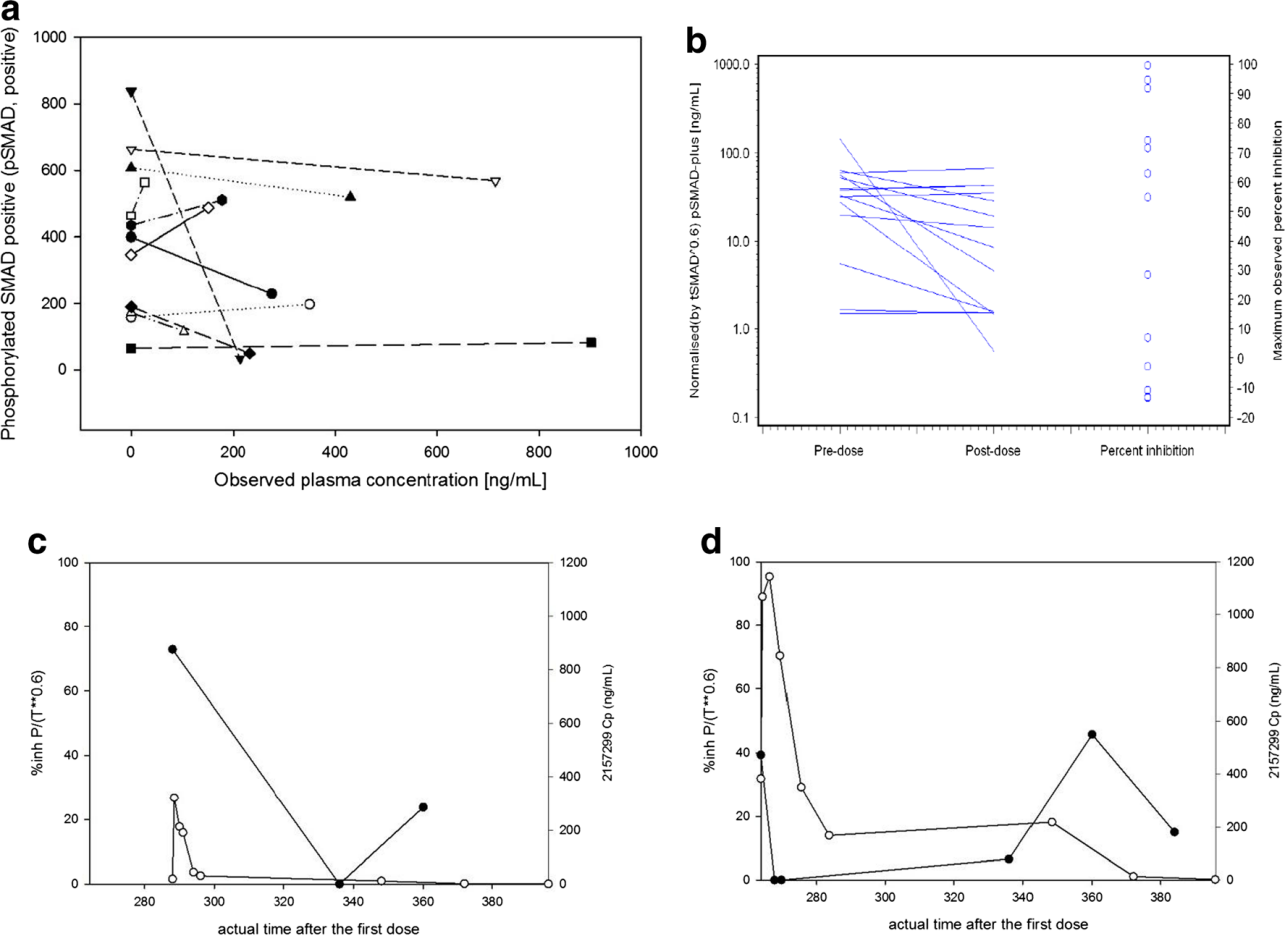
Pharmacodynamic effect of galunisertib. **Panel a:** Pre-dose normalized pSMAD2 vs minimum post-dose pSMAD2 (left axis) and maximum percentage inhibition of normalized pSMAD2 (right axis). Percent inhibition observed in most patients ranged from 10 to 100 % (Part A glioma patients only). **Panel b**: pSMAD2 inhibition is plotted in relationship to concentration on the last day of dosing (Day 14) of the first cycle. **Panel c and d**: pSMAD2 inhibition (solid circles) is shown in relationship to the concentrations (open circles) in hours after galunisertib administration was stopped (336 h = 14 days) for 2 patients (Cohort 1: **panel c,** Cohort 3: **panel d**)

Using an ELISA to detect changes of pSMAD2 in isolated PBMCs as a PD response marker [9], we observed changes after galunisertib administration. Results from Cohort 3 (*n* = 14 patients) were used to confirm reduction of pSMAD2 levels in PBMCs because this cohort had a sufficient number of patients to assess the PK and PD relationship during dose escalation. In 11 of the 14 patients, pSMAD2 inhibition was assessed at the observed maximum concentration for galunisertib about 2–6 h after administration on Day 14 of the first cycle. In 6 of the 11 patients, pSMAD2 levels were reduced in relationship to drug concentration, while in the remaining 5 patients, there was no relationship at the expected C_max_ (Fig. 2a). A reduction in pSMAD2 was observed in 9 (64 %) of the 14 patients during the first 14 days of treatment (Fig. 2b). For 2 patients, this trend was observed in both Cycles 1 and 2 (not shown). Once the drug was stopped after 14 days of administration, pSMAD2 changes were assessed for the next 3 days in 2 patients, one in Cohort 1 and 1 in Cohort 3 (Fig. 2c and d). The pSMAD2 inhibition was still present although drug levels were low or undetectable. The mean of the observed percentage inhibition of normalized pSMAD2^+^ from Cycle 1 Day 1 and Day 12 for Cohort 3 together with the estimated means from the model are provided in Table 2.

### Changes in T cell subsets

**Fig. 3:**
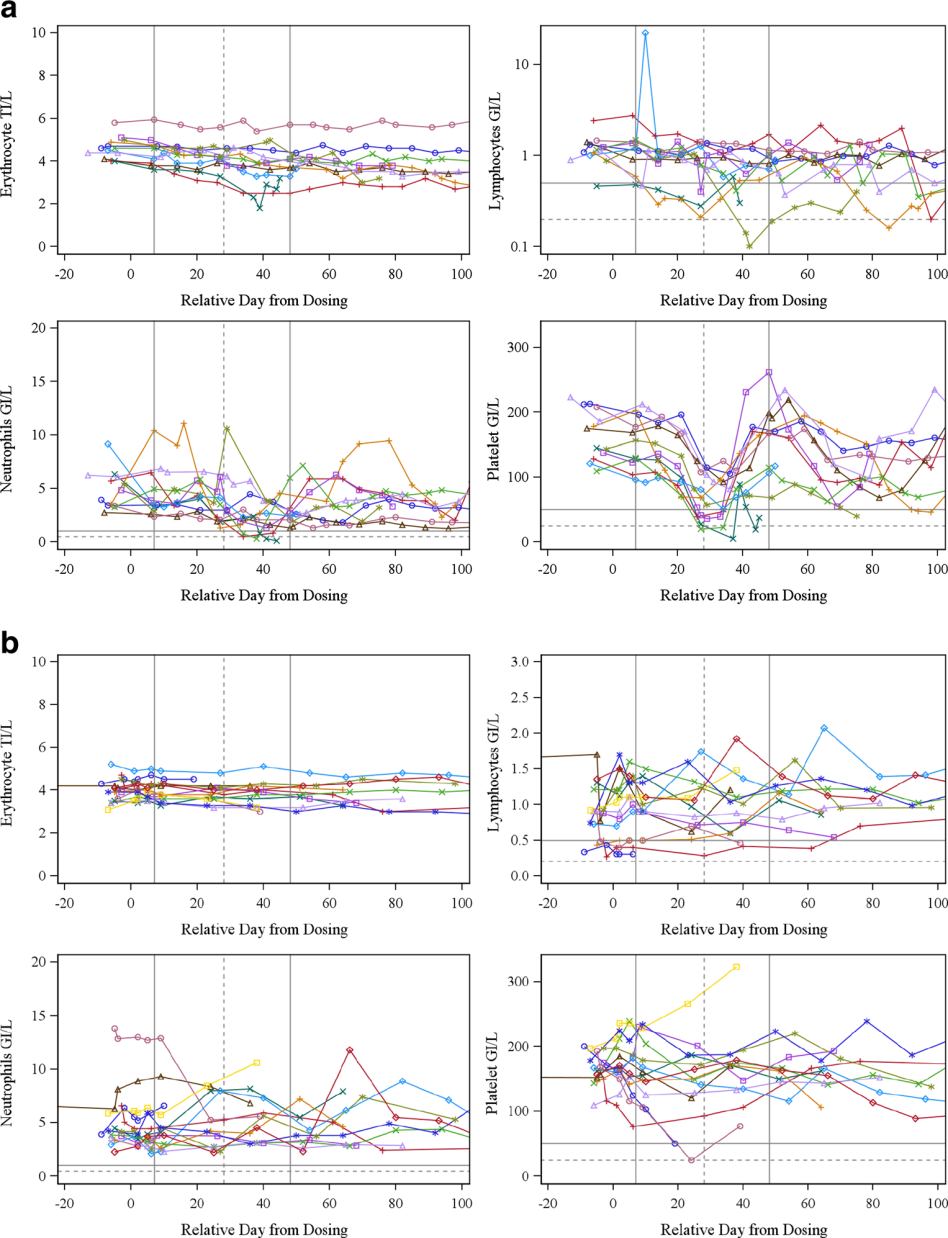
Counts of lymphocytes, erythrocytes, neutrophils and platelets in patients (as represented by *individual lines*) of Part B (combination of galunisertib and lomustine, **panel 3a**) and Part C (monotherapy of galunisertib, **panel 3b**). Lymphocytes were reduced as result of the lomustine treatment compared to Part C (see for comparison the effect on T cell subset examination on Fig. 4). Solid vertical lines indicate galunisertib dosing

An amendment was introduced to assess T cell subsets in Part B and Part C. Some patients receiving the combination of galunisertib and lomustine had a reduction in lymphocyte counts consistent with bone marrow toxicity related to lomustine as evidenced by platelet and neutrophil reduction (Fig. 3a). In contrast, patients in Part C who received more than 2 cycles of treatment showed no comparable bone marrow toxicity as neutrophils and platelets were unchanged (Fig. 3b) and lymphocyte counts were stable. In Part B, lymphocyte counts and the respective subsets were reduced, while in Part C the counts either stabilized or increased for CD3^+^, CD8^+^, CD4^+^, and CD4^+^CD25^+^CD127^−^/LOFoxp3^+^ cells (Fig. 3).

**Fig. 4:**
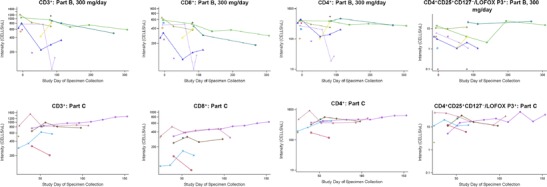
T cell subset assessment after galunisertib treatment in combination with lomustine (Part B) or as monotherapy (Part C). Patients (as represented by *individual lines*) receiving more than 2 cycles of treatment showed a reduction across all T cell subsets if they were treated with the combination of lomustine and galunisertib, but showed stable T cell counts when receiving galunisertib alone

### Baseline tumor tissue evaluation

In order to interpret protein and gene expression as potential prognostic factors we used the concept of clinical benefit based on radiographic response and/or stable disease for ≥6 cycles (Table 3).

**Table 3:** Genetic mutations of variants across >1 sample for patients with low grade glioma/secondary glioblastoma and primary glioblastoma

Patient ID	Tumor Grade	Cycles (n)	IDH1	IDH2	CIC	ATRX	TP53	EGFR	CDKN2A	CDKN2B	CDK4	RB1	NF1	PTEN	PIK3CA	MDM4	PI3KR1
Part A																	
R33	Low	29 (CR)	R132H		R215W												K567E
R23	Secondary	13 (PR)	R132H			K1045fs*1	G245S				Amp						
R16	Primary	22 (PR)											A198fs*7	Y16*			
R19	Primary	46 (CR)					R156G & Q165P						C328fs*1 & R2411fs*10				
R21	Primary	40 (SD)					Y234H					splice					
R34	Low	3	R132H			K626fs*23	R273C										
R28	Low	3						Amp									
R38	Low	3						R108K + Amp	Loss	Loss					H1047R		
R20	Primary	2							Loss	loss				E43fs*9			
R39	Primary	1					L194R							loss			E462fs
R35	Primary	2															
Part B																	
R54	Low	10 (PR)		R172K	A253T		G105S					splice	splice	P96S			
R62	Secondary	12 (SD)	R132G				K305fs*40										
R53	Secondary	2							Loss	Loss					C420R		
R48	Primary	2						A298T& Amp	Loss						R93W	Amp	
R51	Primary	2						Amp	Loss	Loss							
R61	Primary	2						Amp	Loss	Loss							
R64	Primary	4						R324L + Amp		Loss				Loss			
R47	Primary	2						Amp			Amp					Amp	
R50	Primary	2					W91*				Amp						
R52	Primary	1					C242Y	Amp			Amp						

*is a standard nomenclature in the description of mutations fs* means frameshift and *1 means the position of the stop codon

Baseline pSMAD2 expression was assessed in tumor tissue from the original tumor biopsy for 50 patients with glioma by IHC. The majority of the patients had an H-score below 100, and the observed pSMAD2 tumor expression was lower in patients who were treated for ≥6 cycles: H-score median (1st; 3rd quartiles) was 47.5 (12;75) for patients treated for ≥6 cycles and for patients treated for <6 cycles it was 75 (27.5;122.5). However, there was no statistical difference between both groups (*p* = 0.222). Genes were sequenced in 24 samples from patients with glioma in Part A and Part B, of which 21 (21/58; 36 %) had sufficient tumor material (2 samples had no tumor and 1 sample had insufficient material). Gene alteration data were obtained for 11 patients in Part A and 10 patients in Part B (Table 3). On average, there were 4 known/likely functional mutations or 14 variants including alterations of unknown functional significance across 21 tumor samples (data not shown). Of the 21 patients, 7 (33 %) samples were from patients with clinical benefit and 14 (67 %) from those with no clinical benefit. Among these 21 samples, there were 8 from patients with either a secondary glioblastoma or low-grade glioma and 13 with a primary glioblastoma. Genetic alterations were retrospectively evaluated by comparing patients with clinical benefit versus no clinical benefit (CR/PR and SD ≥6 cycles versus those receiving <6 cycles of galunisertib) (Tables 3, 4).

Of the 8 patients with secondary or low-grade glioma, 5 (5/8;62 %) had an isocitrate dehydrogenase (IDH) 1 or IDH2 mutation (Table 3), and 4 of these 5 showed clinical benefit, while none of the 3 low-grade or secondary gliomas without IDH mutations showed radiographic responses or SD ≥6 cycles. Among the 13 patients with primary glioblastoma, none had an isocitrate dehydrogenase IDH1 or IDH2 mutation, and 3 patients (3/13; 23 %) showed radiographic responses or SD ≥6 cycles.

**Table 4:** Summary of treatment responses (no clinical benefit was reported for patients in Part C)

Reasons	Part A *N* = 39 n (%)	Part B *N* = 26 n (%)
Cycles on study treatment, median (range)	2 (1–46)	2 (1–22)
Treatment response*	*n* = 30	*n* = 26
CR/PR (%)	5 (16.7)	2 (7.7)
SD ≥6 cycles	1 (3.3)	4 (15.4)
CR/PR/SD ≥6 cycles	6 (20.0)	6 (23.1)*
SD	10 (33.3)	5 (19.2)
On study treatment at study closure in 2012	2 (6.7)	1 (3.8)
CR/PR/SD ≥6 cycles	6	6*
Primary	3	2
Low grade/secondary glioma	3	4
Treatment Responses by glioma grade and genetic mutation where tumor tissue was available (*n* = 21)		
	IDH1/2	Other
Secondary or low grade glioma (*n* = 8)		
Clinical Benefit	4/8	0/8
No Clinical Benefit	1/8	3/8
Primary glioma (*n* = 13)		
Clinical Benefit	0/13	3/13
No Clinical Benefit	0/13	10/13

Abbreviations: *CR* complete response, *PR* partial response, *SD* stable disease

* Macdonald criteria for all but 1 patient, where RECIST was used

Additionally, the results from this small data set indicate that tumors containing epithelial growth factor receptor (EGFR) variants may not be responsive to galunisertib. All 8 patients with tumors (8/8;100 %) containing EGFR variants (2 in Part A and 6 in Part B) were treated for <6 cycles, while patients with tumors not containing the EGFR variant 54 % (7/13) were treated for ≥6 cycles (Table 3). CDKN2A loss was an additional variant exclusively observed in the nonresponsive tumors. The CDK4 amplification was present in 1 patient who responded, but mainly present in patients who did not have a response or clinical benefit. Thus, having an IDH1/2 variant was associated with a response, while EGFR, CDKN2A, and CDK4 variants were associated with a lack of response.

### Baseline plasma protein evaluation

**Fig. 5:**
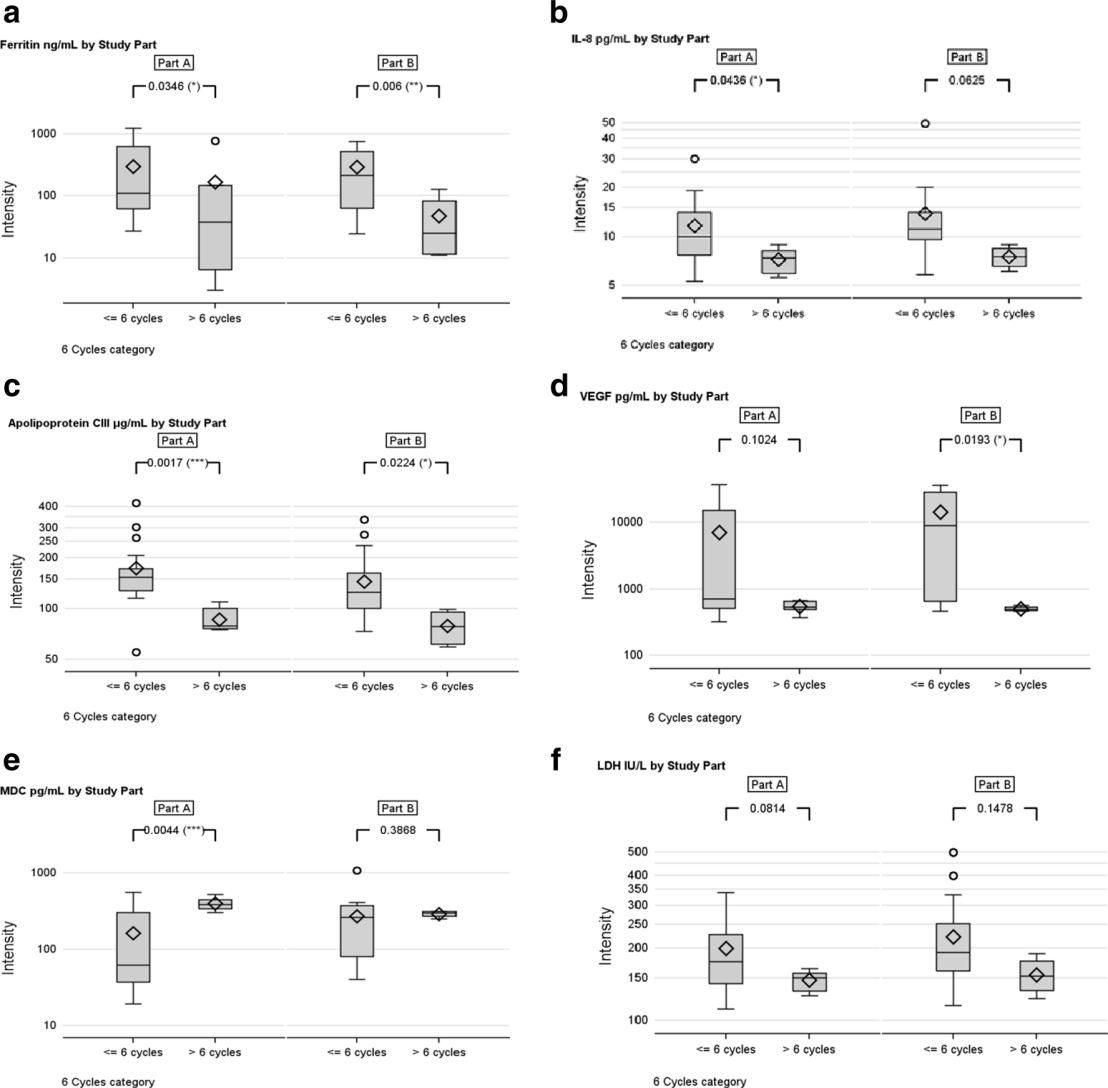
Plasma proteins at baseline. Each panel represents one plasma biomarker displayed for each part (Part A and Part B). Comparison is made between patients who received ≤6 cycles of treatment with those who received ≥6 cycles of treatment. Comparison between both such groups were performed within each part of the study and significance shown on top of the bar graphs (unadjusted p-value): **panel a**: ferritin; **panel b**: IL-8; **panel c**: apolipoprotein CIII; **panel d**: VEGF; **panel e**: MDC/CCL22; **panel f**: LDH

As previously described, an MAIP was used [13] to determine plasma protein levels at baseline. It was expected that some plasma proteins would differentiate patients receiving ≤6 cycles from those receiving >6 cycles of galunisertib treatment (Fig. 5). In patients of Part A and Part B, ferritin (panel a), IL-8 (panel b), apolipoprotein (panel c), vascular endothelial growth factor (VEGF) (panel d), and lactate dehydrogenase (LDH) (panel f) were lower in patients who were treated for >6 cycles and elevated in patients treated for ≤6 cycles. Only macrophage-derived chemokine (MDC or CCL22 [chemokine (c-c motif) ligand-22]) levels (panel e) were higher at baseline for patients treated with galunisertib longer than 6 cycles.

## Discussion

Galunisertib treatment showed single-agent activity in patients with glioma who had progressed on treatments that were previously effective. Determined by radiographic responses or durable disease stabilization for at least 6 cycles of treatment, clinical benefit was seen in 12 of 79 (15 %) patients, all in glioma. The responses also are reminiscent of those reported for patients with glioma treated with trabedersen [5]. Similar to trabedersen, the responses appeared to be more common in patients with lower WHO grade glioma. Secondary or lower-grade gliomas are commonly associated with IDH1 mutations [14, 15], and IDH1 mutations have been recently associated with TGF-β signaling [16]. To further investigate this possibility, we evaluated the remaining tumor tissue for IDH1/2 mutation. Among the 5 patients with an IDH1/2 mutated tumor, there were 4 with either radiographic response or SD ≥6 cycles. One patient with IHD1/2 mutation had no clinical benefit (CR, PR and SD ≥6 cycles). Besides these patients with IDH1/2 mutation, 3 other patients with primary glioma benefited from galunisertib. Therefore, it is possible that TGF-β signaling is enriched in the IDH1/2 population but is also present in other subgroups of glioma. One such overarching phenotype may be characterized by the mesenchymal activation pathway [17].

Galunisertib affected the pSMAD2 expression in PBMCs. Generally, percentage inhibition (compared to pre-dose) of pSMAD2 concentrations in PBMCs were more variable than expected ([10]) and was higher than the variability used to size this study to detect a 50 % pSMAD2 inhibition. Nevertheless, reductions post-dose were observed in 64 % of patients in Cohort 3, and mean percentage inhibition was estimated to be approximately 40 % at the end of 14 days of treatment. There are some observations suggesting that an indirect relationship between the exposure and pSMAD2 inhibition may exist which is supported by pSMAD2 inhibition continuing after the drug is stopped. Overall, the reduction of pSMAD2 in PBMCs appears to be consistent with observations in galunisertib-treated animals (data not shown) and ex vivo studies with human PBMCs or purified T cells [10, 18] and suggests that the drug has the intended pharmacological activity in patients.

In addition to these pharmacological effects in PBMCs, we assessed the changes in inhibitor of DNA-binding protein (ID1) and CD44 expression in pre- and post-treatment tumor tissue of 1 patient who underwent surgical re-resection of his brain tumor during the off-period of the intermittent dosing-regimen [4]. After treatment with galunisertib, the expression of both ID1 and CD44 was reduced, suggesting that TGF-β signaling was inhibited in the glioma tissue.

Because galunisertib likely also affects the TGF-β signaling in T regulatory cells [19], we used the T cell subsets as another PD marker for response. However, we did not observe a change in the T cell subsets as described for ipilimumab, which also targets T regulatory cells [20]. The lack of changes in T regulatory cells during the monotherapy with galunisertib does not preclude the possibility that changes are occurring in T cells. This is supported by the changes in pSMAD2 levels in PBMCs from patients receiving galunisertib. Hence, functional immune monitoring will be needed to better characterize the effect of galunisertib on immune cells.

Among several plasma proteins, we observed that patients with high baseline levels of MDC/CCL22 received more than 6 cycles of treatment in both Part A and Part B. The post-treatment levels of MDC/CCL22 did not show a change (data not shown). MDC/CCL22 is associated with modulating T regulatory cells [21, 22] and it may be involved in the activation of antigen-presenting cells. Whether such levels confer a better prognosis remains to be assessed. The lack of a change in MDC/CCL22 may perhaps indicate that this chemokine is not affected by galunisertib, but long-term examinations will be needed to further confirm this initial impression. Other plasma proteins were also found to be at different levels for patients treated with ≥6 cycles or ≤6 cycles. Their patterns of baseline levels were consistent with previous reports, suggesting that they were associated with prognosis in glioma, including LDH [23], apolipoprotein CIII [24], ferritin [25], IL-8 [26], and VEGF [27].

The protein expression of pSMAD2 in tumor tissue was assessed to determine whether high pSMAD2 expression was present in this group of patients with glioma as previously reported [28, 29]. Patients with longer treatment cycles had lower baseline expression of pSMAD2 in their tumors. Whether pSMAD2 levels can be used as a future prognostic or predictive marker is confounded by the following factors: First, pSMAD2 expression may change over the course of the disease similar to other tumor-associated markers in secondary glioma [30]. Since the pSMAD2 staining was done only on the initial diagnostic tissue and not on tissue before treatment with galunisertib, the extent of TGF-β pathway activation prior to the galunisertib treatment is unknown. For example, radiotherapy is known to increase TGF-β expression [31, 32] and thus the subsequent pSMAD2 expression in patients could have increased during first-line treatment with chemoradiation. Second, the location of pSMAD2 expression is possibly related to prognosis. In our study, we did not differentiate pSMAD2 staining based on its anatomical location. Using the same antibody as in the present study, high expression in parenchymal cells was prognostic for survival, while perivascular staining was not [29]. Future assessment of pSMAD2 staining will have to include the differential evaluation of the staining patterns.

In summary, galunisertib has a predictable PK and a favorable safety profile to continue its clinical investigation in Phase II studies and has shown clinical benefit at the recommended dose predicted by a preclinical PK/PD model.
